# Supplementary material for: Communities in C. elegans connectome through the prism of non-backtracking walks
Source: Sci Rep. 2023 Dec 21;13:22923. doi: 10.1038/s41598-023-49503-5 (PMC10739864; doi:10.1038/s41598-023-49503-5)
Supplement: Supplementary file 1 — Supplementary Information. [file 41598_2023_49503_MOESM1_ESM.pdf]

Supplementary Information
Communities in C.elegans connectome through the prism of non-backtracking walks

Arsenii A. Onuchin, Alina V. Chernizova, Mikhail A. Lebedev, Kirill E. Polovnikov

In this document, we provide 5 Supplementary Figures and 1 Table that additionally illustrate the results of the clustering analysis of the *C.elegans* structural connectome from the main text. In the first two figures (Figs. S1, S2) we discuss the technical performance of the flow matrix algorithm of clusterization and the properties of its eigenvalues spectrum. In the next figure (Fig. S3) we provide pairwise intersections between the clusters, found by three spectral methods: Normalized Laplacian, Modularity matrix and Flow matrix. In the following two figures (Figs. S4, S5) we provide an additional biological justification to the found clusters. The supplementary table contains information about the pairwise overlaps between various structural clusters and biological modules. All the data used in the paper is deposited on this link: Google Spreadsheet.

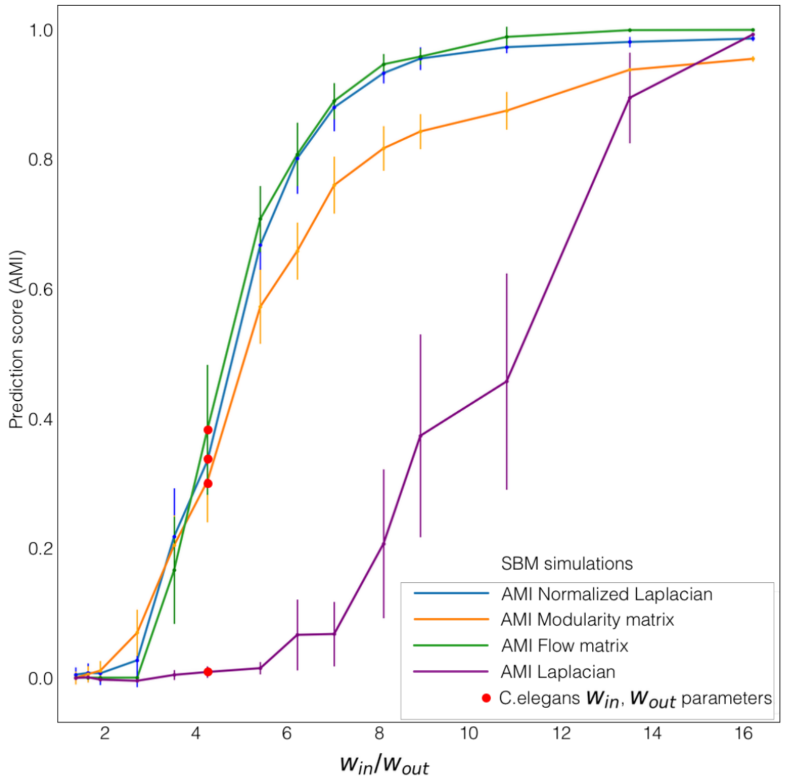

FIG. S1: Mean Adjusted Mutual Information (AMI) score for simulations with the planted Stochastic Block Model (SBM) with weight parameters w_{in}, w_{out}, size $N = 279$ and number of clusters $k = 7$. The AMI score is averaged over 200 realizations of random SBM graphs with fixed parameters (the error bars reflect the corresponding statistical error). For each realization of a random graph AMI score is computed between the underlying SBM partition (the ground truth) and clusters inferred in that graph by four different operators: Laplacian, normalized Laplacian, modularity operator and flow matrix. The red dots denote the empirical value $w_{in}/w_{out} = 0.22/0.05 \approx 4.4$, corresponding to the connectome (data from Chen et al, 2006 [34,35]).

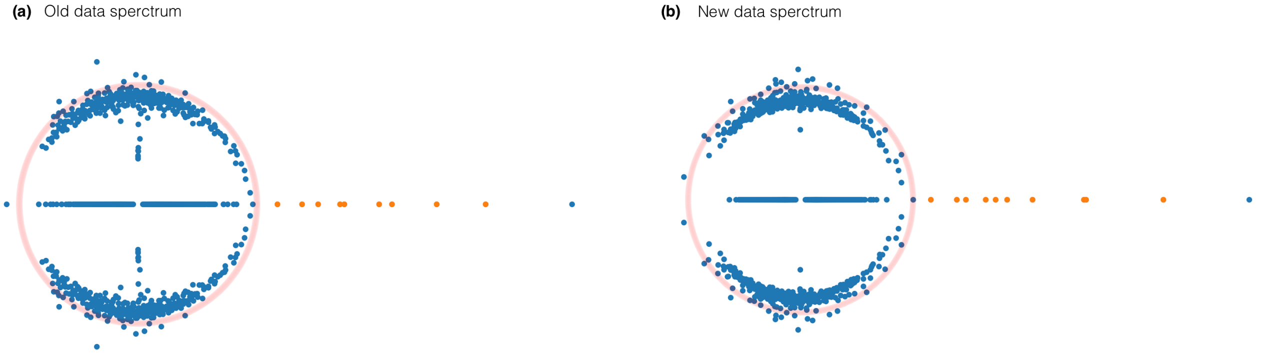

FIG. S2: Comparison of the eigenvalues spectra of the flow matrix computed for two datasets: **(a)** Chen et al, 2006 [34, 35] ("old data") and **(b)** Cook et al, 2019 [25] ("new data "). In both cases the spectrum consists of complex eigenvalues constrained within a circle of radius r (see Eq. (5) of the main text) and a set of isolated eigenvalues on the real axis (orange) together with the leading eigenvalue (blue). Despite the 2-fold increase of the total number of edges in the new dataset, the amount of isolated eigenvalues remains the same, $k = 10$.

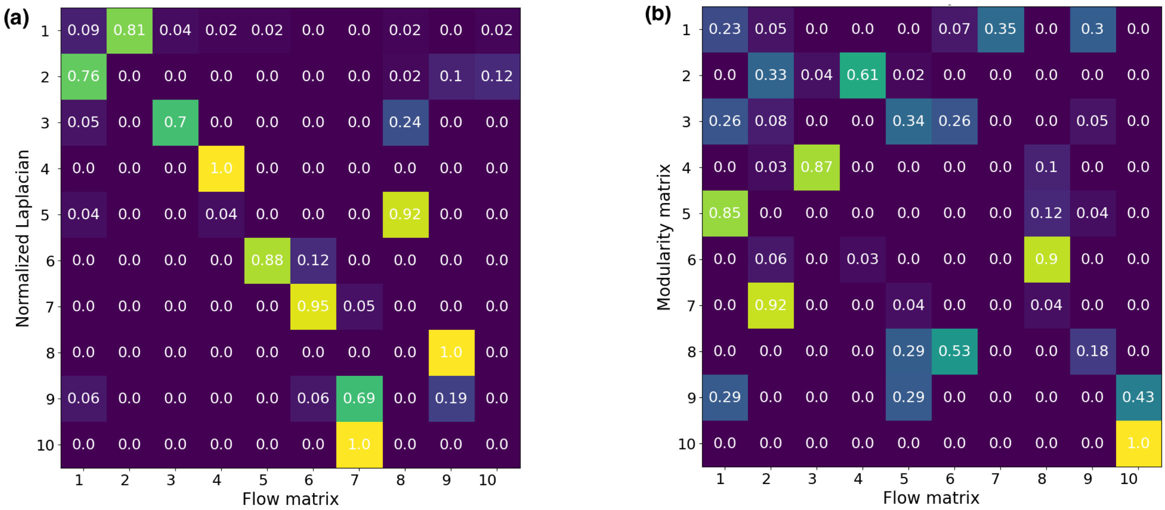

FIG. S3: This figure shows the percent of the cluster, which was obtained by Normalized Laplacian or Modularity matrix spectral methods, contained in the corresponding Flow matrix cluster. (**a**) Overlaps between Normalized Laplacian and Flow matrix 10 clusters found in the new connectome data [25] (**b**) Overlaps between Modularity matrix and Flow matrix 10 clusters found in the new connectome data [25].

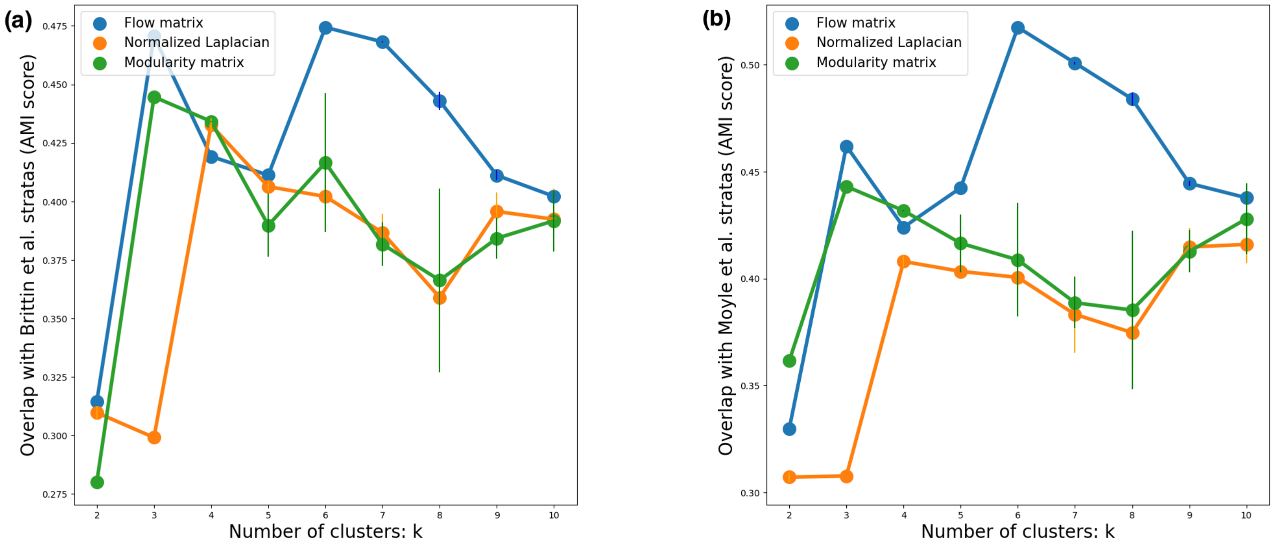

FIG. S4: (a) The AMI score computed between the modules from Brittin et al. [29] and partitions of the structural connectome (the new data, Cook et al, 2019 [25]) into k clusters, as inferred by three operators: flow matrix, normalized Laplacian and modularity matrix. The peak of AMI score for the flow matrix corresponds to $k = 6$ modules identified in Brittin et al. [29]. (b) The same as in (a) but for $k = 6$ modules from Moyle et al. [30].

	AMI score
IMMA clusters [41] vs Ganglia (old data [34, 35])	0.31
IMMA clusters [41] vs Moyle et al. [30] (old data [34, 35])	0.43
IMMA clusters [41] vs Brittin et al. [29] (old data [34, 35])	0.40
ERMM clusters [33] vs Ganglia (old data [34, 35])	0.34
ERMM clusters [33] vs Moyle et al. [30] (old data [34, 35])	0.36
ERMM clusters [33] vs Brittin et al. [29] (old data [34, 35])	0.41
IMMA clusters [41] vs ERMM clusters [33] (old data [34, 35])	0.41
Flow Matrix clusters (maximum for 8 clusters) vs Ganglia (new data [25])	0.425
Flow Matrix clusters vs Ganglia (old data [34, 35])	0.34
Flow Matrix clusters (maximum for 6 clusters) vs Moyle et al. [30] (new data [25])	0.51
Flow Matrix clusters (maximum for 6 clusters) vs Brittin et al. [29] (new data [25])	0.47
Flow Matrix clusters vs IMMA clusters [32] (old data [34, 35])	0.4
Flow Matrix clusters vs ERMM clusters [33] (old data [34, 35])	0.45

TABLE S1: The overlap (AMI score) values between modules found by various algorithms in the old [34, 35] and new [25] C.elegans connectomes (as indicated) and biological benchmarks (ganglia, contactome modules [29, 30]).

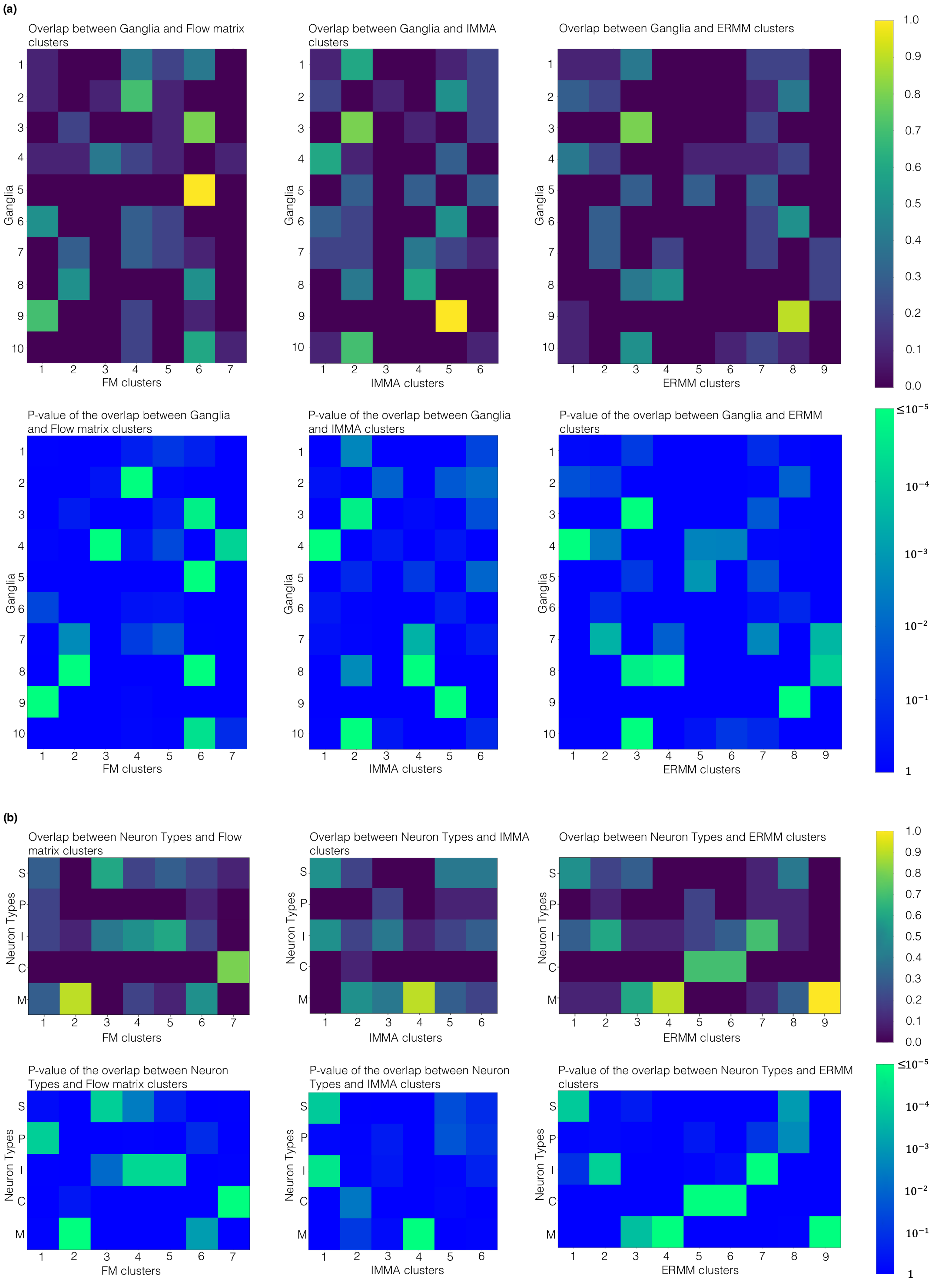

FIG. S5: (**a**) Overlaps and p-values between ganglia and clusters found by three different algorithms: flow matrix (FM), iterative modularity maximization algorithm (IMMA) [41] and Erdos-Renyi Mixture Model (ERMM) [33]. The structural clusters are obtained on the old connectome data [34, 35]. (**b**) Overlaps and p-values between the clusters (same as in (a)) and neuronal types: sensory neurons (S), polymodal neurons (P), interneurons (I), command neurons (C), motoneurons (M).
